# Supplementary material for: How pore formation in complex biological membranes is governed by lipid composition, mechanics, and lateral sorting
Source: PNAS Nexus. 2025 Feb 21;4(3):pgaf033. doi: 10.1093/pnasnexus/pgaf033 (PMC11879431; doi:10.1093/pnasnexus/pgaf033)
Supplement: pgaf033_Supplementary_Data [file pgaf033_supplementary_data.pdf]

Supplementary Information of: How pore formation in complex biological membranes is governed by lipid composition, mechanics, and lateral sorting

Leonhard J. Starke,[†] Christoph Allolio,[‡] and Jochen S. Hub[*,†]

[†]*Theoretical Physics and Center for Biophysics, Saarland University, Saarbrücken, Germany*

[‡]*Faculty of Mathematics and Physics, Charles University, Prague, Czech Republic*

E-mail: jochen.hub@uni-saarland.de

Supplementary Methods

Setup of membrane systems

Atomistic models of the following complex membranes were set up: (a) asymmetric model of a mammalian plasma membrane,[1] (b) asymmetric models of the outer and (c) inner mitochondrial membranes,[2] symmetric models of (d) the mammalian Golgi apparatus, (e) the lysosome, (f) endosomes,[2] (g) yeast endoplasmic reticulum (ER),[3] and (h) a symmetric model composed of *E. coli* lipid extract.[4] In addition, to dissect the roles of lipid head groups and tails on the free energy landscape of pore formation, model membranes with simple composition were set up. Single-component membranes were set up containing 16:0-18:1-phosphatidylcholine (POPC), -phosphatidylethanolamine (POPE), -phosphatidylinositol (POPI),

-phosphatidylserine (POPS), -phosphatidic acid (POPA), or -phosphatidylglycerol (POPG). Moreover, membranes were set up with binary mixtures of POPC plus cholesterol, 16:0-lyso-PC, oleic acid, or 18:0-24:0-sphingomyelin. For each lipid composition, a small membrane patch with 81 to 162 lipids per leaflet was set up to simulate pore nucleation. Larger membrane patches with 300 to 342 lipids were set up to simulate pore expansion. The lipid compositions of all simulation systems are listed in Tables S2–S9.

Symmetric membranes were generated using MemGen.[5] Structures of small asymmetric systems of mammalian inner and outer mitochondrial membranes were taken from the pre-equilibrated membranes described in Ref. 2. The other asymmetric membrane systems (large mitochondria membranes and small/large plasma membranes) were built from the corresponding symmetric membrane models using the following protocol. i) The lipid coordinates of the upper and lower monolayer were extracted from the corresponding equilibration simulations of the outer leaflet and inner leaflet based symmetric membranes from a frame within the last 30 ns of the trajectory to ensure that the membrane areas were converged. ii) The two monolayers were placed on top of each other and their center-of-mass distance was adjusted to avoid atomic overlaps. After energy minimization with the steepest descent algorithm for 500 steps, the systems were solvated and water molecules located between the membrane leaflets were removed. Neutralizing K^+ ions were added. iii) Two short (200 ps) initial equilibration simulations were conducted using 2 fs or 4 fs time steps, respectively.

Recently it has been shown that asymmetric membranes may exhibit significant differential stress between the two membrane leaflets.[6] Here, we calculated the lateral pressure profile for the asymmetric plasma membrane model to obtain the differential stress. Details of pressure calculations are described below. We found that the plasma membrane exhibits differential stress among the two leaflets with tension values of only ∼6 pN/nm. These results confirm the low stress values given by Doktorova *et al.*[1] Furthermore, because the membrane area hardly changes during pore nucleation,[7] we expect that differential stress, even if present, would play only a minor role during pore nucleation. During pore expansion

where the membrane area expands, differential stress may rapidly relax by lipid by flip-flop across the open pore.

Lipid topologies and structures were taken from the CHARMM-GUI web server[8,9] except for *E. coli* polar lipids, which were kindly provided by the authors of Ref. 4, as well as the parameters for ether-PE (PLQS) and OAdaPE, which were kindly provided by Edward Lyman.

Atomistic MD simulations were set up and carried out using GROMACS[10] together with the CHARMM36 force field for lipids.[11–15] All systems were neutralized with K^+Cl^- ions and solvated with the CHARMM-modified TIP3P water,[16] which involves Lennard-Jones interactions of hydrogen atoms. The systems were equilibrated for 100 ns. The temperature was controlled at 300 K using the velocity rescale thermostat.[17] The pressure was kept at 1 bar using semi-isotropic exponential pressure relaxation with a stochastic term (c-rescale).[18] Coulomb interactions were calculated using the particle-mesh Ewald method.[19] Lennard-Jones interactions were treated according to the CHARMM specifications, using a cut off at 1.2 nm with the forces being gradually switched off between 1.0 and 1.2 nm. Hydrogen bonds of water molecules were constrained using SETTLE[20] while all other bonds involving hydrogen atoms were constrained using LINCS.[21] Hydrogen mass repartitioning[22] with the default factor of 3 was applied throughout, thus a time step of 4 fs was used for all simulations.

Reaction coordinate for pore formation

Potentials of mean force (PMF) of pore formation were computed using umbrella sampling (US)[23] along a joint reaction coordinate (RC) ξ_p for pore nucleation and pore expansion.[24] The RC is implemented into an in–house modified version of GROMACS, freely available at `https://gitlab.com/cbjh/gromacs-chain-coordinate`. The RC ξ_p is an extension of the chain coordinate ξ_ch proposed in Refs. 25,26. For graphical illustrations of ξ_p and ξ_ch we refer to Ref. 24. The chain coordinate ξ_ch has been designed to track pore nucleation and quantifies the degree of connectivity of a polar transmembrane defect. ξ_ch is defined

using a membrane-spanning narrow cylinder partitioned into N_s slices of thickness d. The coordinate evaluates the fraction of slices that are occupied by polar atoms and thus take values in $\xi_\mathrm{ch}\in[0,1)$ with $\xi_\mathrm{ch}\approx 1$ indicating a fully formed transmembrane defect. The following parameters were used to specify ξ_ch: Both the positions of water oxygen atoms and lipid phosphate moieties were used to compute ξ_ch. The radius R_cyl of the cylinder was set to 0.9 nm in this work. The choice of R_cyl ensures that the defect is localized in the membrane plane but does not control the radius of the defect. If R_cyl is too large, two laterally displaced partial defects connected to the upper and lower water reservoirs could be misinterpreted as a single continuous membrane-spanning defect, which could lead to hysteresis problems. The length of the cylinder $N_\mathrm{s}\cdot d$ is given by the thickness of the slices (here set to $d=0.1\,\mathrm{nm}$) and the number of slices N_s. N_s was selected for each membrane such that $\xi_\mathrm{ch}\approx 0.25$ was obtained for a flat unperturbed membrane, implying that approximately 25% of the slices (at the head group regions) were filled by polar atoms. The values of N_s used in this study are listed in Table S10.

Because ξ_ch projects larger pores onto $\xi_\mathrm{ch}=1$ it is not suitable for studying pore expansion. Pore expansion is instead characterized by the approximated radius R of the fully formed pore leading to a joint RC ξ_p for pore nucleation and pore expansion,[24] as recently used to obtain the free energy landscape of membrane electroporation.[27,28] The RC ξ_p is defined as follows:

$$\xi_\mathrm{p}(\mathbf{r})=\xi_\mathrm{ch}(\mathbf{r})+H_\epsilon\left[\xi_\mathrm{ch}(\mathbf{r})-\xi_\mathrm{ch}^s\right]\frac{R(\mathbf{r})-R_0}{R_0}. \tag{1}$$

Here, $\mathbf{r}$ are the Cartesian coordinates of the system. The pore radius $R(\mathbf{r})$ is computed from the polar atoms within a layer of thickness D within the hydrophobic core of the membrane (here set to $D=1.0\,\mathrm{nm}$ for all systems except for the asymmetric and outer leaflet composition of the plasma membrane where $D=1.5$ nm was used) by assuming a cylindrical shape of the defect and a volume per polar atom of $v_0=0.02996\,\mathrm{nm}^3$, corresponding to the molecular volume of water. R_0 corresponds to the radius of a minimal transmembrane pore. The value of R_0 depends on the membrane system and takes values between 0.32 nm

and 0.44 nm (Table S10). H_ϵ denotes a smoothed differentiable variant of the Heaviside step function, which switches from 0 to 1 within the interval $[-\epsilon, \epsilon]$ (here set to $\epsilon = 0.05$). The value ξ_{ch}^s determines where to switch from pore nucleation to expansion, here set to $\xi_{\text{ch}}^s = 0.925$. Hence, for $\xi_{\text{ch}} > \xi_{\text{ch}}^s + \epsilon$ corresponding to a fully formed transmembrane defect, we have $H_\epsilon = 1$ and $\xi_{\text{ch}} \approx 1$ and, according to Eq. 1, ξ_{p} is given by the radius R of the open pore in units of R_0 (i.e., $\xi_{\text{p}}(\mathbf{r}) \approx R(\mathbf{r})/R_0$). For $\xi_{\text{ch}} < \xi_{\text{ch}}^s - \epsilon$, we have $H_\epsilon = 0$ and the coordinate ξ_{p} is equivalent to ξ_{ch}, thus quantifying the degree of connectivity of the transmembrane defect. For more details on the definition and implementation of ξ_{ch} and ξ_{p} we refer to previous work.[24–26]

Umbrella sampling simulations of pore nucleation and expansion

PMFs of pore formation involve a nucleation phase and an expansion phase. In this study, we carried out two sets of umbrella sampling (US) simulations using either ξ_{ch} or ξ_{p}, and we combined the US windows into a single PMF. Starting points for umbrella sampling were generated using constant-velocity pulling simulations along ξ_{ch} from 0 to 1 over 100 ns for pore nucleation simulations and along ξ_{p} from 0 to 7 over 200 ns for pore expansion. A force constant of 3000 kJ/mol was used. For pore expansion simulations, flat-bottomed restraints along the membrane normal were applied to the lipid C2 atoms (in the glycerol region). The thickness of the flat-bottom region R_{fb} was chosen to allow for normal head group fluctuations, but to exclude large-scale membrane undulations or deformations (Table S10). The atoms used for making the membrane whole prior to computing the membrane center of mass (periodic boundary condition atoms) were chosen near the tip of the lipid tails at the center of the membrane.

For US simulations of pore nucleation along ξ_{ch}, a spacing of 0.08 was used between US windows in the early nucleation regime ($\xi_{\text{ch}} < 0.64$) with a force constant of 5000 kJ/mol and a spacing of 0.02 was used close to the nucleation barrier with a force constant of 10000 kJ/mol. For simulations of pore expansion along ξ_{p}, a spacing of 0.03 was used around

the pore nucleation barrier $\xi_p \in (0.64, 1.12)$ with a force constant of 5000 kJ/mol, while for larger pores a spacing of 0.15 was used with a force constant of 400 kJ/mol. For pore nucleation, 27 US windows were run for 150 ns each. For pore expansion, 63 US windows were run for 50 ns for single-lipid membranes, 100 ns for binary lipid mixtures and 150 ns for complex membranes. The temperature during US simulations was kept at 310 K. For analysis of the nucleation simulations the initial 20 ns were discarded for equilibration. In case of the expansion simulations of single lipid membranes the initial 10 ns were discarded while for all membrane systems containing multiple lipid species the initial 50 ns were removed. US windows from pore nucleation in the range $\xi_{ch} < 0.875$ were combined with the US windows of pore expansion to compute PMFs spanning both nucleation and expansion. PMFs were calculated using the GROMACS implementation of the weighted histogram method (WHAM).[29,30] Uncertainties of the PMFs were estimated by Bayesian bootstrapping of complete histograms over 50 rounds.[30]

The free energy of pore nucleation ΔG_{nuc} was defined as the value of the PMF at $\xi_p = 0.92$, characterized by the presence a thin transmembrane water wire. The uncertainty of ΔG_{nuc} was taken from the uncertainty of the PMF. The line tension γ along the pore rim was taken from the slope of a linear fit to the PMF in the range $4 \leq \xi_p \leq 6.5$. The fit was performed for a set of 50 bootstrapped PMF profiles thus resulting in a set of γ_i $i \in [1, 50]$. γ was then defined as the arithmetic mean $\langle\gamma_i\rangle$ and its uncertainty was therefore characterized by the standard deviation $\sigma(\gamma_i)$.

Convergence of PMFs

PMF calculations along complex conformational transitions frequently suffer from poor convergence or hysteresis effects. To exclude that our PMFs are strongly biased by such problems, we computed PMFs in, both, forward and in backward direction by taking starting frames for umbrella sampling from constant-velocity pulling simulations along either pore-opening or pore-closing pathways. This analysis was carried out for the membranes of the

Golgi apparatus, lysosome, endosome, and for the outer leaflet composition of the plasma membrane. Pulling simulations were carried out for 200 ns and US windows were sampled for 150 ns each, except for the plasma system for which we extended the US simulations to 300 ns each. The PMFs are shown in Figure S1 and ΔG_{nuc} and γ value in Table S1, demonstrating that hysteresis effects are relatively small. For the pore nucleation phase, a slightly increased hysteresis effect of 9 kJ/mol is only observed for the Golgi membrane (Fig. S1a). We explain this effect by the presence of positively curved lyso-PC (that strongly stabilizes the pore rim) in the Golgi membrane, rendering exhaustive sampling of the lipid composition at the pore rim particularly challenging.

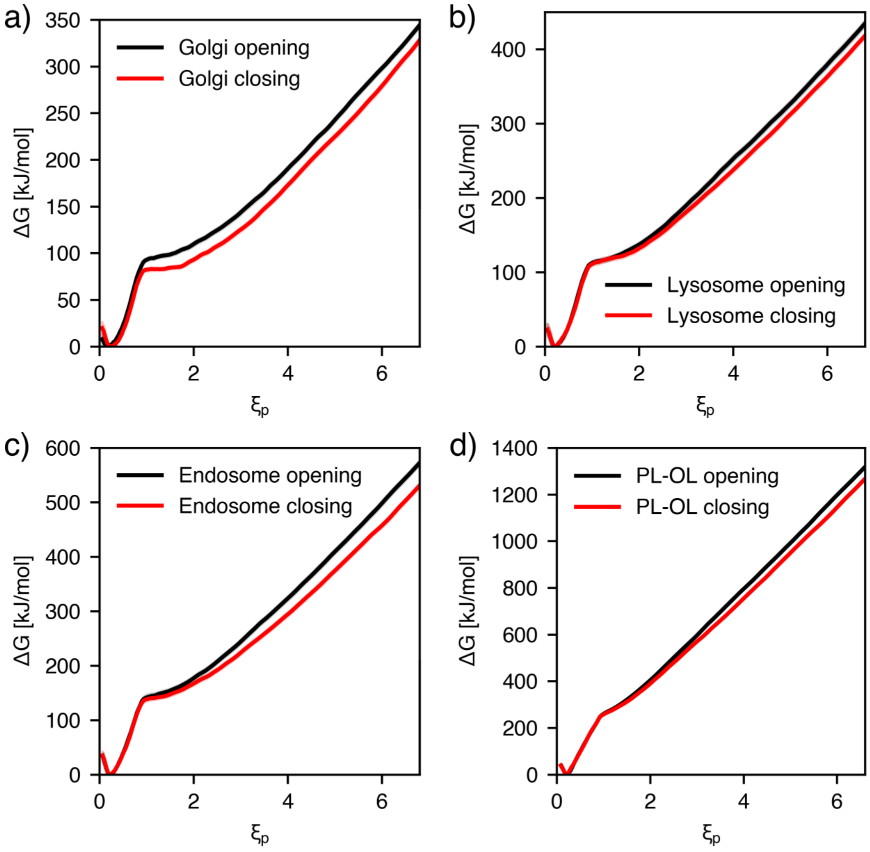

Figure S1: Comparison of PMFs along the opening (black) or closing pathway (red) for (a) the membrane of the Golgi apparatus, (b) lysosome, (c) endosome, and (d) for a membrane based on the lipid composition of the outer plasma membrane leaflet. Opening and closing PMFs were obtained by starting umbrella sampling simulations from MD frames taken either from constant-velocity pulling simulations in opening or closing direction, respectively.

Table S1: ΔG_{nuc} and γ values from PMF calculations along pore opening and closing pathways for four complex biological membranes, revealing only a minor degree of hysteresis.

	$\Delta G_{\text{nuc,opening}}$ [kJ/mol]	$\Delta G_{\text{nuc,closing}}$ [kJ/mol]	γ_{opening} [pN]	γ_{closing} [pN]
Golgi	90 ± 2	81 ± 2	39.6 ± 0.2	39.1 ± 0.2
Lysosome	109 ± 3	107 ± 3	48.8 ± 0.4	47.6 ± 0.3
Endosome	138 ± 5	134 ± 4	69.7 ± 0.4	65.5 ± 0.4
Plasma-OL	246 ± 3	243 ± 4	138.0 ± 1.0	134.4 ± 0.8

Additional control simulations

Influence of flat-bottomed restraints

As described above, flat-bottomed restraints along the membrane normal z were used during pore expansion simulations to avoid simulation instabilities that may arise from large-scale membrane deformations (Table S10). To exclude that the flat-bottomed restraints would bias the PMF considerably, we carried out two additional simulations of a POPC membrane with (i) looser restraints by increasing R_{fb} from 2.0 nm to 2.2 nm or (ii) tighter restraints by decreasing R_{fb} to 1.8 nm. We found that, upon decreasing R_{fb} to 1.8 nm, γ increases by $\sim$3 pN to 35.3±0.4 pN (see Table S13), possibly because the restraints disfavor the relaxation of that the toroidal pore shape near the pore rim. In contrast, upon increasing R_{fb} to 2.2 nm, γ decreases only slightly to 31.0 ± 0.5 pN. Thus, we used R_{fb}=2.0 nm in this study because it yields numerically highly stable simulations while imposing only a small effect on γ as compared to simulations with looser restraint.

Validation of the protocol of merging PMFs from nucleation and expansion by comparison with a protocol involving freezing the lateral cylinder position at small ξ_p positions

In our implementation of the chain coordinate,[25] the lateral position of the membrane-spanning cylinder is by default dynamically defined, thereby allowing the cylinder to follow the defect as the defect travels in the membrane plane. This feature is critical to avoid undesirable hysteresis effects near the free energy barrier of pore formation (if present).[25,26]

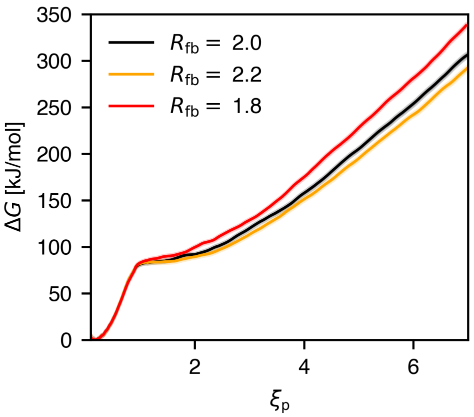

Figure S2: Comparison of PMFs for a POPC membrane using different radii R_{fb} for the flat-bottomed restraints.

However, using a laterally mobile cylinder during the early stages of pore nucleation across a large membrane system leads to an overestimation of the pore free energy due to rapid lateral movement of the cylinder and, consequently, imprecise integration of the equations of motion. Two solutions have been conceived to solve this problem: (i) In Ref. 24, the lateral cylinder position was frozen at $\xi_{\text{p}} < 0.7$ until a partial water defect has formed, thus enabling a stable lateral cylinder position in all umbrella windows. (ii) In the present study, we merged umbrella histograms from a small membrane patch during pore nucleation — where problems owning to such rapid lateral movement of the cylinder do not occur— with a large membrane patch used during pore expansion. To test the validity of the approach adopted in this study, we compared PMFs of pore formation for the ER membrane from protocol (i) (Figure S3a,b, purple lines) with PMFs from protocol (ii) (Figure S3a,b, blue lines).

For reference, and as expected, the PMF obtained with a mobile cylinder at all ξ_{p} (Figure S3a,b, orange) overestimates the the pore free energy considerably as compared to PMFs obtained with protocols (i) and (ii), in agreement with previous findings.[24] We therefore discourage the use of a mobile cylinder at all ξ_{p} for large membrane systems. Critically, the offset between PMFs from protocols (i) and (ii) equals 8 kJ/mol, in reasonable agreement with the value obtained owing to the loss of lateral pore entropy upon laterally freezing the

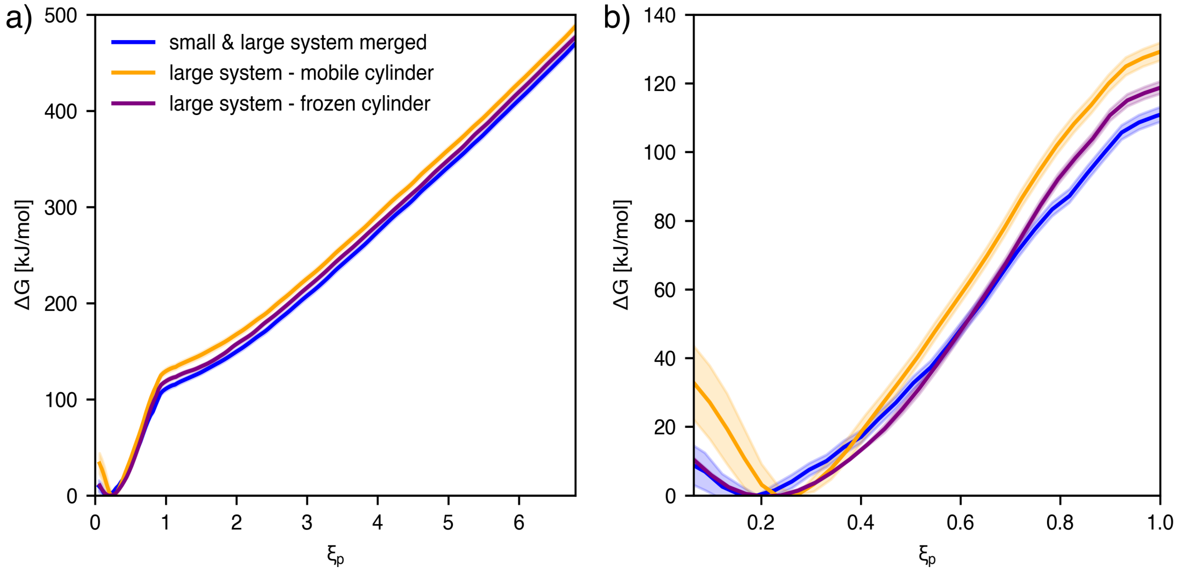

Figure S3: On the effect of laterally freezing the membrane-spanning cylinder at $\xi_\mathrm{p} < 0.7$ versus computing the PMFs by merging umbrella histograms from a small and a large simulation system, here analyzed for the ER membrane. (a) PMFs of pore nucleation and expansion and (b) close-up view on the nucleation regime. Blue: PMF from merging umbrella histograms of the small and large simulation systems. Orange: PMF from using a mobile cylinder at all ξ_p. Purple: PMF of pore nucleation and expansion with a laterally frozen cylinder position for $\xi_\mathrm{p} < 0.7$. Statistical errors from bootstrapping are indicated as shaded areas.

cylinder, as given by $k_BT \ln(A_\mathrm{small}/A_\mathrm{cyl}) \approx 6.5\,\mathrm{kJ/mol}$, where A_small denotes the membrane area of the small system and A_cyl the cross section area of the cylinder. Thus, protocols (i) and (ii) yield consistent results, yet yielding pore free energies for either one pore per area A_small or one pore per area A_cyl. Notably, upon translating the pore free energies to probabilities of pore formation for macroscopic membranes (see Main Text), the offset in pore free energy owing to different areas per pore cancel out.

Together, this analysis demonstrates that merging umbrella histograms from small and large systems is a valid protocol to construct the PMF of pore nucleation and pore expansion. For future simulations of pore formation in large membrane systems, however, we recommend the use of a laterally frozen cylinder up to $\xi_\mathrm{p} < 0.7$ for large systems because it is technically simpler compared to the protocol of merging histograms from small and large simulation system.

Additional simulation analysis

To analyze the distributions of lipids along the rim of an open pore, lipid densities were computed as function of radial distance r from the pore center and of membrane normal z. Lipid densities were calculated from simulations restrained at $\xi_\mathrm{p} \approx 5.5$ over 400 ns for complex membranes and over 200 ns for simpler model membranes, respectively. For visualization purposes the thickness parameter D used to define ξ_p was increased to 1.5 nm. The density distributions were obtained using an in–house modified version of the GROMACS module gmx density available at `https://gitlab.com/cbjh/gromacs-chain-coordinate`.

The thickness of the membrane core d_hc was derived from the mass density profiles of the hydrophobic tail atoms, which was computed from the last 50 ns of the equilibration simulations. The thickness was then defined as the width of the density profile at a density threshold of 500 kg/m^3.

Extraction of elastic properties

For the computation of elastic properties, pre-equilibrated bilayers of the small systems (see above) were continued for 600 ns using Gromacs 2020.3.[10] The temperature was maintained at 303.15 K using a Nosé-Hoover thermostat,[31,32] and the pressure was maintained at 1 bar using a semi-isotropic Parrinello-Rahman barostat.[33] We used a 1.2 nm real-space cutoff for electrostatic and Lennard-Jones interactions. Long-range electrostatics were treated using the particle-mesh Ewald method.[19] Because the pressure decomposition does not support the SETTLE algorithm,[20] the triangular geometry of water was constrained using LINCS with order five.[21] No dispersion corrections or potential switching were applied. The timestep was 2 fs. 75.000 MD snapshots with velocities were taken from each simulation.

The local stress tensor was computed using the rerun functionality of the gmx mdrun module of our implementation[34] of a Goetz-Lipowsky-Decomposition[35] into the code by Sega *et al.*[36] The modified GROMACS version is available at `https://github.com/allolio`. The same cutoffs were applied as described above. Our approach is similar to the method in Ref.

37. Specifically, we set the normal pressure p_N to impose zero surface tension:

$$\sigma = \int_{-l}^{l} \pi(z)\,\mathrm{d}z = \int_{-l}^{l} \Big[-\frac{1}{2}\{p_{xx}(z) + p_{yy}(z)\} + p_\mathrm{N}\Big]\,\mathrm{d}z = 0. \tag{2}$$

Here, σ is obtained from the diagonal components of the stress tensor,[38] the integral is carried out over the entire simulation box $[-l, l]$ along the membrane normal z, where $z = 0$ corresponds to the center of the bilayer. Since this approach requires p_N being close to 1 bar, we validated that that deviations from this target were ≤ 3.2 bar for all simulations.

Bending modulus κ and tilt modulus κ_θ were computed using the ReSIS method.[39] We used lipid director definitions and an implementation from the the LIPIDATOR-TOOLKIT available at `https://github.com/allolio/lipidator-toolkit`. The director vectors were chosen following the protocol described in Johner *et al.*[40] In Khelashvili *et al.*[41] it was shown that the exact choice of the atoms used in the director definitions has only a small effect on the results. The director vector was computed using the last three carbon atoms of each chain and several head group heavy atoms. These director definitions are compatible with the definitions used in earlier publications.[40,41] The cholesterol director was also chosen following earlier conventions.[41] These choices allowed the use of the well-known[42,43] relation of the first bending moment to the product of spontaneous curvature, J_s and κ

$$\kappa J_\mathrm{s} = \int_{0}^{l} \pi(z) z\,\mathrm{d}z. \tag{3}$$

Here, the integration is carried out along the membrane normal $\hat{\mathbf{e}}_z$ and only over one monolayer, and hence monolayer elastic properties are used. The reported values are averages over both monolayers. Bilayer values can be obtained by summing up the monolayer values, resulting in a bilayer $J_\mathrm{s}^\mathrm{b} = 0$ for symmetric membranes. We used spline interpolation for the integration of the pressure profiles. Values computed specifically for this study are for membranes of POPA, POPG, POPI, or POPS as well as for mixtures of POPC with 16-lyso-PC, oleic acid, or cholesterol. Other data were taken from previous work.[34,37,44] New director

vectors for oleic acid and lyso-PC will be added to the LIPIDATOR-TOOLKIT repository.

The pressure tensor was computed from 600 ns with 75,000 snapshots, whereas κ and κ_θ were computed from 200 ns and 25,000 snapshots. Error bars denote standard errors computed from subsampling. For the pressure tensor, nine windows of 66.6 ns length each were used. For κ and κ_θ, ten windows of 20 ns were used, yielding standard errors in the order of $\sim 1\,k_BT$, in line with Doktorova *et al.* for mixed systems.[1] The error of κJ_s was propagated into the error of J_s using Gaussian error propagation. We are aware that long-wavelength fluctuations may lead to lateral sorting, which may affect the average bending rigidity,[45] however, such effects do not apply in the small systems examined here.

To calculate the differential stress for the asymmetric plasma membrane model, the lateral pressure profile of the small cyto+ model membrane[1] was calculated following the procedure outlined above using 200 ns and 25000 MD frames. The tension for each leaflet was calculated via integration of the pressure profile for the upper or lower leaflet, respectively.

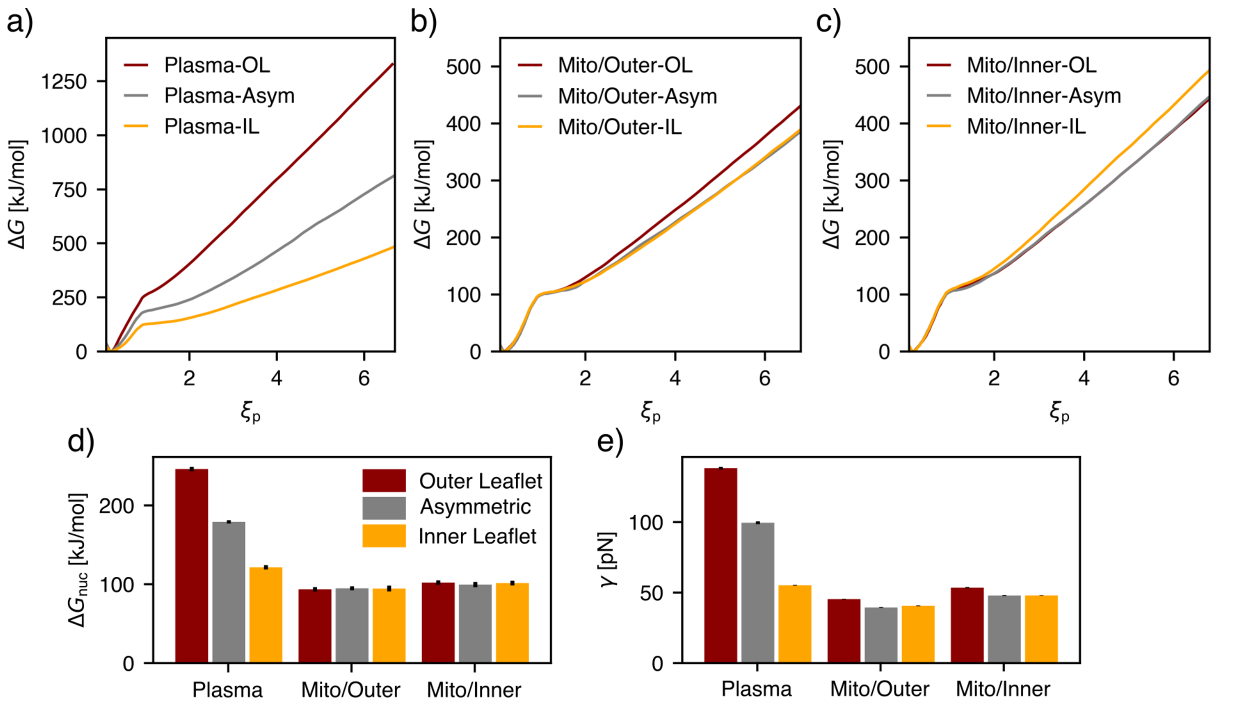

Figure S4: PMFs of pore formation for symmetric versus asymmetric membrane models. PMFs for membranes based on the composition of the outer leaflet (OL, dark red) and inner leaflet (IL, orange) are shown together with the profile of the corresponding asymmetric membranes (gray): (a) Plasma membranes, (b) outer mitochondrial membrane, and (c) inner mitochondrial membranes. (d) Bar plot of nucleation free energy ΔG_{nuc} and (e) line tension γ. Error bars of ΔG_{nuc} and γ (hardly visible) obtained from bootstrapping.

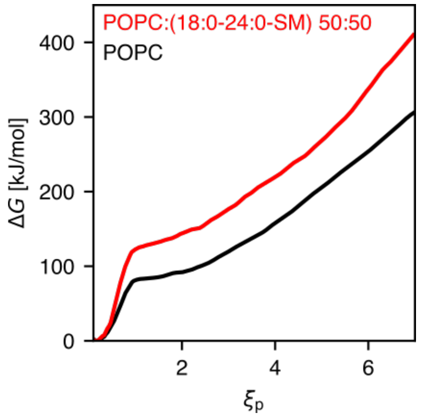

Figure S5: On the large effect of saturated sphingomyelin (18:0-24:0-SM) on pore free energy. PMF for a pure POPC membrane (black) and for a 50:50 mixture of POPC with 18:0-24:0-SM (red).

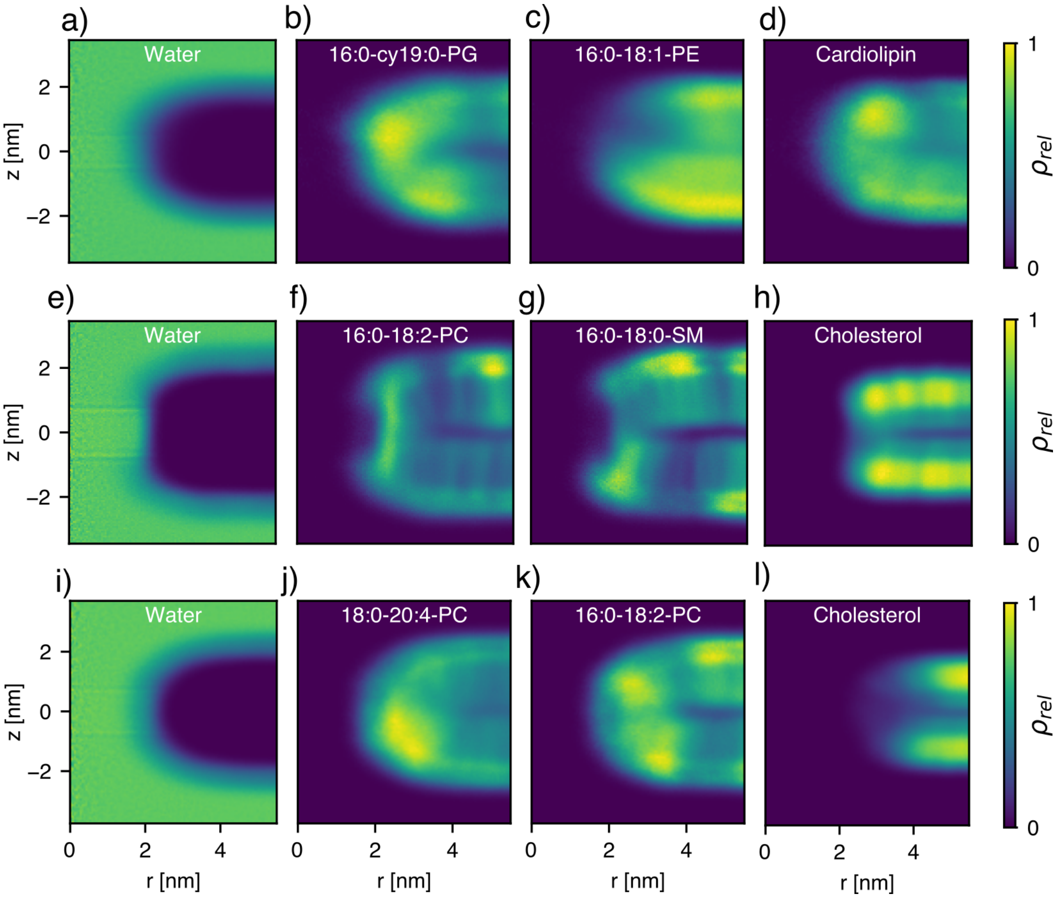

Figure S6: Relative mass densities of water and lipid species along the pore rim, plotted as function of lateral distance r from the pore center and normal distance z from the membrane center. Densities were averaged from extended simulations of umbrella windows with pore radii of approximately 2 nm. Densities are normalized by dividing by the maximal density of the respective constituent. Densities are shown for selected lipids (see labels) for membranes of (a-d) *E. coli* extract ($\xi_p \sim 5.7$), (e-h) outer plasma membrane leaflet ($\xi_p \sim 5.4$), and (i-l) endosome ($\xi_p \sim 5.6$). Evidently, cholesterol and PE lipids are depleted at the pore rim (panels c, h, l), whereas PG lipids (panel b) and –to a lower degree– polyunsaturated PC (panels j) are enriched at the rim.

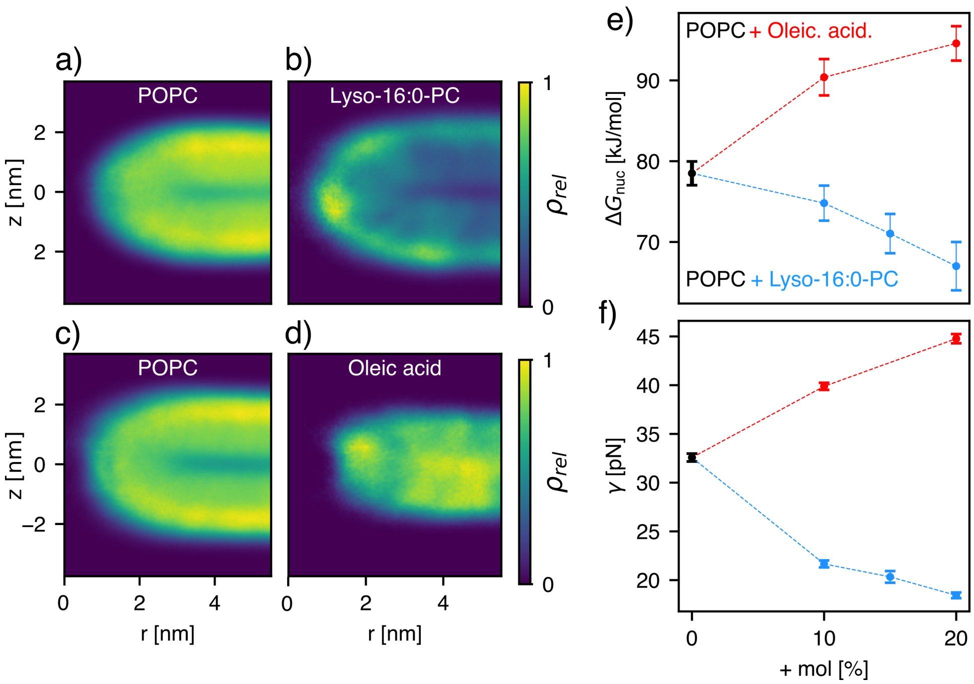

Figure S7: Influence of addition of curvature-inducing lipids. (a-d) Normalized density of lipids along the pore rim as function of radial distance r from the pore center and normal distance z from the membrane center for (a/b) simulation of POPC–lyso-16:0-PC 80:20 and (c/d) POPC–oleic acid 80:20. Lyso-PC and oleic acid are enriched or depleted at the pore rim, respectively. (e) Effect of increasing oleic acid or lyso-PC concentration on ΔG_{nuc} and (f) γ.

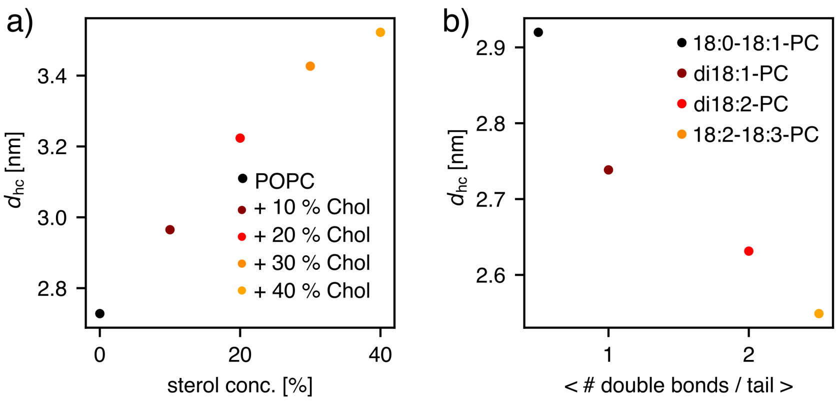

Figure S8: Effect of unsaturation and sterol content on the thickness of the membrane core d_{hc} of the studied model systems. (a) Effect on d_{hc} due to the addition of cholesterol within a POPC membrane. (b) Effect of the degree of unsaturation on d_{hc} using a set of PC model membranes.

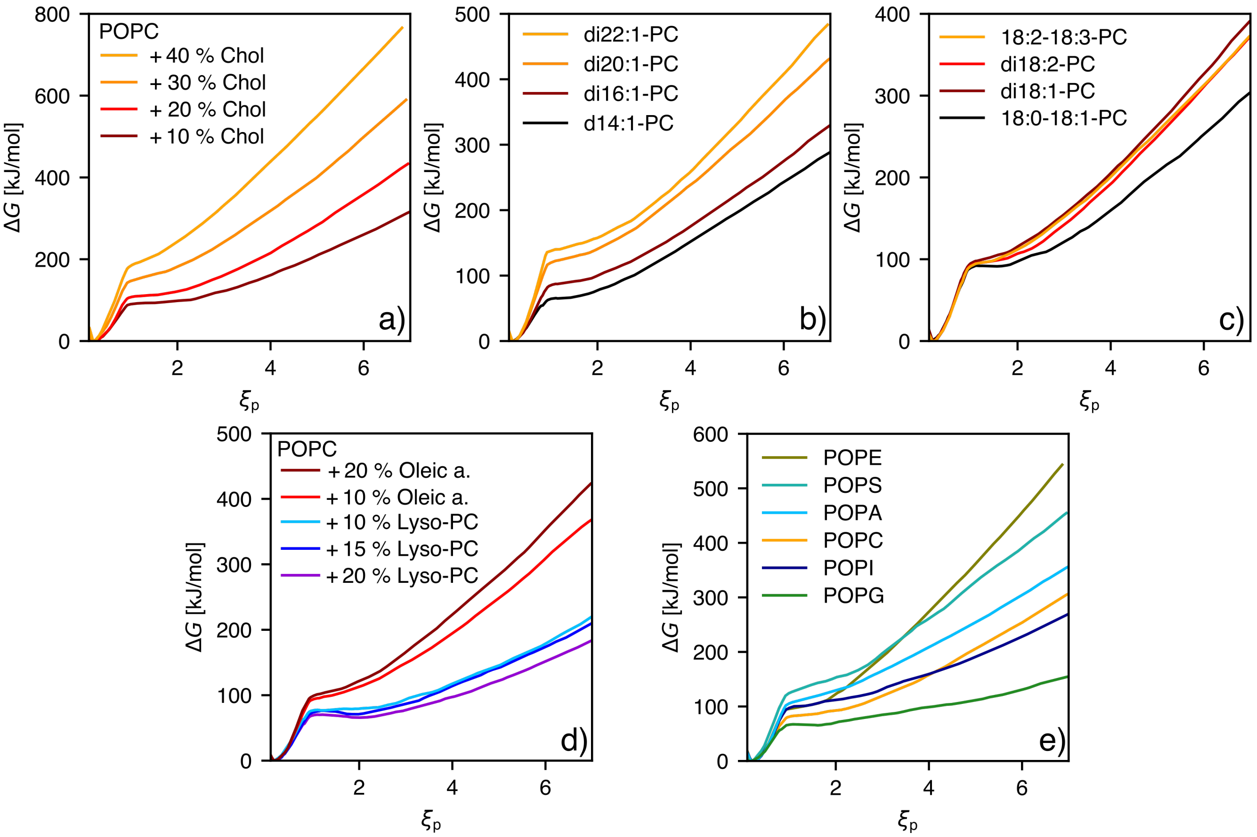

Figure S9: PMFs of model membranes with: (a) with varying sterol content, (b) with varying number of acyl tail carbons, (c) with varying number of double bonds, (d) with varying content of lyso-PC or oleic acid, (e) with varying head group composition.

Table S2: Lipid composition of plasma membrane systems of small (N_{small}) and large (N_{large}) membrane systems. Composition for each the outer leaflets (OL) and inner leaflets (IL)

Component	OL		IL	
	N_{small}	N_{large}	N_{small}	N_{large}
16:0-18:1-PC (POPC)	-	-	8	16
16:0-18:2-PC (PLPC)	20	40	20	40
18:0-18:1-PC (SOPC)	10	20	-	-
16:0-22:4-PC (PAPC)	6	12	-	-
18:1-16:0-SM (PSM)	18	36	-	-
18:1-24:1-SM (NSM)	14	28	-	-
18:1-24:0-SM (LSM)	12	24	-	-
18:0-22:4-etherPE (PLQS)	-	-	20	40
16:0-22:6-PE (PDoPE)	-	-	18	36
18:1-22:4-PE (OAdaPE)	-	-	8	16
16:0-22:4-PS (PAPS)	-	-	30	60
Cholesterol	95	190	35	70
Sum	175	350	139	278

Table S3: Lipid composition of endosomal membrane of the small (N_{small}) and large (N_{large}) simulation systems.

Component	N_{small}	N_{large}
16:0-18:2-PC (PLPC)	8	30
18:0-22:4-PC (SAPC)	14	51
18:0-22:6-PC (SDPC)	5	18
16:0-18:2-PE (PLPE)	2	6
16:0-22:6-PE (PDoPE)	4	12
18:0-22:4-PE (SAPE)	6	21
18:0-22:4-PI (SAPI)	5	18
18:0-22:6-PI (SDPI)	2	6
18:0-24:0-SM (DSM)	7	24
18:1-18:1-SM (OSM)	7	24
Cholesterol	21	90
Sum	81	300

Table S4: Lipid composition of lysosomal membranes of the small (N_{small}) and large (N_{large}) simulation systems.

Component	N_{small}	N_{large}
16:0-18:2-PC (PLPC)	11	33
18:0-22:4-PC (SAPC)	18	53
18:0-22:6-PC (SDPC)	6	18
16:0-18:2-PE (PLPE)	7	21
18:0-22:4-PE (SAPE)	18	53
18:0-22:4-PI (SAPI)	6	18
18:0-22:6-PI (SDPI)	2	6
18:0-22:4-PS (SAPS)	2	6
18:0-22:6-PS (SDPS)	1	3
18:1-16:0-SM (PSM)	3	9
18:1-24:0-SM (LSM)	3	9
18:1-18:1-BMP (BMGP)	7	17
Cholesterol	18	53
Sum	102	300

Table S5: Lipid composition of ER membranes of the small (N_{small}) and large (N_{large}) simulation systems.

Component	N_{small}	N_{large}
16:0-18:1-DAG (POGL)	4	12
18:1-18:1-DAG (DOGL)	3	9
16:1-16:1-PE (DYPE)	17	51
16:1-16:1-PC (DYPC)	20	60
16:0-18:1-PC (POPC)	5	15
16:0-18:1-PI (POPI)	25	75
16:0-18:1-PA (POPA)	2	6
18:1-18:1-PA (DOPA)	1	3
16:0-18:1-PS (POPS)	2	6
18:1-18:1-PS (DOPS)	1	3
Ergosterol	10	30
Sum	100	300

Table S6: Lipid composition of Golgi membranes of the small (N_{small}) and large (N_{large}) simulation systems.

Component	N_{small}	N_{large}
16:0-18:1-PC (POPC)	11	33
16:0-18:2-PC (PLPC)	14	42
18:0-22:4-PC (SAPC)	20	60
16:0-18:2-PE (PLPE)	4	12
16:0-18:0-PE (PSPE)	8	24
18:0-22:4-PE (SAPE)	5	15
16:0-18:0-PI (PSPI)	2	6
16:0-18:1-PI (POPI)	5	15
18:0-22:4-PI (SAPI)	2	6
16:0-18:1-PS (POPS)	4	12
18:0-22:0 -SM (TSM)	12	36
Lyso-16:0-PC	5	15
Cholesterol	8	24
Sum	100	300

Table S7: Lipid composition of outer mitochondrial membranes for outer leaflet (OL) and inner leaflet (IL), each of the small (N_{small}) and large (N_{large}) simulation systems.

Component	OL		IL	
	N_{small}	N_{large}	N_{small}	N_{large}
16:0-18:1-PC (POPC)	6	18	6	18
16:0-18:2-PC (PLPC)	18	54	18	54
18:0-22:4-PC (SAPC)	30	90	30	90
16:0-18:1-PE (POPE)	4	12	2	6
16:0-18:2-PE (PLPE)	9	27	5	15
18:0-22:4-PE (SAPE)	25	75	13	39
18:0-22:4-PI (SAPI)	4	12	22	66
18:0-22:4-PS (SAPS)	1	3	3	9
16:0-18:1-PA (POPA)	1	3	1	3
18:2-18:2/18:2-18:2/CL (TLCL1)	2	4	-	-
Sum	100	300	100	300

Table S8: Lipid composition of inner mitochondrial membranes for outer and inner leaflets.

Component	OL		IL	
	N_{small}	N_{large}	N_{small}	N_{large}
16:0-18:1-PC (POPC)	7	21	3	9
16:0-18:2-PC (PLPC)	19	57	10	30
18:0-22:4-PC (SAPC)	32	96	16	48
16:0-18:1-PE (POPE)	5	15	4	12
16:0-18:2-PE (PLPE)	8	24	8	24
18:0-22:4-PE (SAPE)	24	72	24	72
18:0-22:4-PE (SAPI)	5	15	6	18
18:0-22:4-PE (SAPS)	3	9	3	9
18:2-18:2/18:2-18:2/CL (TLCL1)	11	33	26	78
Sum	114	342	100	300

Table S9: Lipid composition of *E. coli* polar lipid extract of the small (N_{small}) and large (N_{large}) simulation systems.

Component	N_{small}	N_{large}
16:1-16:0/cy17:0-cy19:0-CL (YPMNCL)	1	3
16:1-16:0/cy17:0-18:1-CL (YPMVCL)	1	3
cy17:0-16:0/16:0-18:1-CL (MPPVCL)	2	6
17:0-16:0/16:0-17:0-CL (MPPMCL)	1	3
16:0-cy19:0-PG (PMPG)	10	36
16:0-cy19:0-PG (PNPG)	2	6
16:1-18:1-PG YOPG	1	3
16:0-18:1-PG (POPG)	4	12
16:0-16:1-PG (PYPG)	2	6
16:1-18:1-PE (YOPE)	12	45
16:0-cy17:0-PE (PMPE)	15	54
16:0-cy19:0-PE (PNPE)	5	18
cy17:0-18:1-PE (MVPE)	7	27
16:0-18:1-PE (POPE)	17	77
Sum	81	300

Table S10: Parameters of pore reaction coordinate for different systems. Number of slices of transmembrane cylinder used to define ξ_{ch} during umbrella sampling of pore nucleation N_s^{nuc} and during pore expansion N_s^{exp}, radius of the flat-bottomed region R_{fb} of the flat-bottomed potential used to exclude large-scale membrane undulations, and radius R_0 of a thin nucleated pore used to define ξ_p. R_0 is not a free parameter but set automatically at the beginning of the simulation based on the number of slices (N_s^{nuc} or N_s^{exp}) and D, see Ref. 24.

System	N_s^{nuc}	N_s^{exp}	R_{fb} [nm]	R_0 [nm]
Plasma-Asym	42	42	2.2	0.344646
Plasma-OL	44	45	2.2	0.386685
Plasma-IL	39	38	2.1	0.353815
E. coli extract	35	34	2.0	0.372404
Endosome	41	43	2.1	0.335857
ER	29	37	2.0	0.358956
Lysosome	37	38	2.0	0.353815
Golgi	34	36	2.0	0.363457
Mito/Outer-OL	31	32	2.0	0.380517
Mito/Outer-IL	31	31	2.0	0.383527
Mito/Outer-Asym	33	33	2.0	0.374414
Mito/Inner-OL	32	35	2.0	0.365406
Mito/Inner-IL	33	35	2.0	0.365406
Mito/Inner-Asym	33	34	2.0	0.372404
(16:0-18:1)-PC (POPC)	25	27	2.0	0.39796
(16:0-18:1)-PA (POPA)	32	33	2.0	0.374414
(16:0-18:1)-PE (POPE)	34	34	2.3	0.372404
(16:0-18:1)-PI (POPI)	30	32	2.0	0.380517
(16:0-18:1)-PG (POPG)	23	22	2.0	0.426354
(16:0-18:1)-PS (POPS)	32	32	2.3	0.380517
POPC:Chol 90:10	27	32	2.3	0.380517
POPC:Chol 80:20	34	31	2.3	0.383527
POPC:Chol 70:30	37	37	2.3	0.358956
POPC:Chol 60:40	40	42	2.3	0.344646
POPC:Lyso-16:0-PC 80:20	22	25	2.0	0.406944
POPC:Lyso-16:0-PC 85:15	22	28	2.0	0.396042
POPC:Lyso-16:0-PC 90:10	23	26	2.0	0.38848
POPC:Oleic acid 80:20	30	31	2.3	0.38848
POPC:Oleic acid 90:10	28	28	2.3	0.38848
POPC:(18:0-24:0)-SM 50:50	27	30	2.3	0.38848
14:1-14:1-PC (DRPC)	19	19	1.7	0.438885
16:1-16:1-PC (DYPC)	24	27	1.9	0.39796
18:1-18:1-PC (DOPC)	28	29	2.0	0.390468
20:1-20:1-PC (DGPC)	32	35	2.2	0.365406
22:1-22:1-PC (DEPC)	38	39	2.6	0.354824
12:0-12:0-PC (DLPC)	14	16	1.7	0.461476
18:0-18:1-PC (SOPC)	25	32	2.0	0.380517
18:2-18:2-PC (DUPC)	26	29	2.0	0.390468
18:2-18:3-PC (LLPC)	26	28	2.0	0.396042

Table S11: Results of linear regression $Y = m \cdot X + n$ to estimate correlation between ΔG_{nuc} and γ with elastic/geometric properties.

Y	X	m	n	R
ΔG_{nuc}	$\gamma_{\text{Helf}}^{\text{bend}}$	0.50 ± 0.19	50 ± 12	0.59
γ	$\gamma_{\text{Helf}}^{\text{bend}}$	0.54 ± 0.05	-4 ± 3	0.97
ΔG_{nuc}	d_{hc}	88 ± 16	-160 ± 50	0.92
γ	d_{hc}	73 ± 15	-170 ± 50	0.89
ΔG_{nuc}	κ_θ	1.08 ± 0.13	30 ± 7	0.96
γ	κ_θ	0.80 ± 0.27	-9 ± 13	0.71

Table S12: Experimental measurements of line tension γ reported previously.

membrane composition	γ [pN]	Reference
Egg PC	14.2 ± 0.7	Portet T. & Dimova R.A. 2010[46]
Egg PC	8.6 ± 0.4	Chernomordik L. *et al.* 1985[47]
DOPC	28 ± 3	Portet T. & Dimova R.A. 2010[46]sun2022physical
DOPC from Avanti	20 ± 4	Karatekin E. *et al.* 2003[48]
DOPC from Sigma	6.9 ± 0.4	Karatekin E. *et al.* 2003[48]
E.coli PE	16.0 ± 0.6	Chernomordik L. *et al.* 1985[47]
SOPC	9.2 ± 0.7	Zhelev D.V. *et al.* 1993[49]
DOPG/DOPE (4/6)	15.6 ± 0.7	Tazawa K. & Yamazaki M. 2023[50]
DOPG/DOPC (4/6)	10.7 ± 0.4	Tazawa K. & Yamazaki M. 2023[50]
DOPG/DOPC/LPC (40/55/10)	6.7 ± 0.2	Tazawa K. & Yamazaki M. 2023[50]
POPC	40 ± 6	Lira R.B. *et al.* 2021[51]
POPC/POPG (1/1)	23 ± 6	Lira R.B. *et al.* 2021[51]

Table S13: Pore nucleation free energies and line tension from US simulations of pore opening for all simulated membrane systems.

System	ΔG_{nuc} [kJ/mol]	γ [pN]
Plasma-Asym	180 ± 4	100.0 ± 1.0
Plasma-OL	246 ± 3	138.0 ± 1.0
Plasma-IL	121 ± 3	55.2 ± 0.3
E. coli extract	105 ± 4	51.4 ± 0.2
Endosome	138 ± 5	69.7 ± 0.4
ER	106 ± 2	50.8 ± 0.5
Lysosome	109 ± 3	48.3 ± 0.4
Golgi	90 ± 2	39.6 ± 0.2
Mito/Outer-OL	94 ± 4	40.6 ± 0.3
Mito/Outer-IL	94 ± 3	45.3 ± 0.3
Mito/Outer-Asym	95 ± 3	39.4 ± 0.4
Mito/Inner-OL	102 ± 3	53.5 ± 0.5
Mito/Inner-IL	102 ± 4	45.0 ± 0.2
Mito/Inner-Asym	100 ± 4	48.0 ± 0.4
16:0-18:1-PC (POPC)	79 ± 2	32.6 ± 0.4
16:0-18:1-PA (POPA)	102 ± 2	34.6 ± 0.6
16:0-18:1-PE (POPE)	93 ± 6	65.5 ± 0.7
16:0-18:1-PI (POPI)	94 ± 2	25.0 ± 0.4
16:0-18:1-PG (POPG)	63 ± 3	11.1 ± 0.5
16:0-18:1-PS (POPS)	120 ± 3	45.4 ± 0.5
POPC:Chol 90:10	88 ± 2	35.0 ± 0.5
POPC:Chol 80:20	104 ± 2	50.4 ± 0.7
POPC:Chol 70:30	142 ± 3	68.3 ± 0.8
POPC:Chol 60:40	177 ± 4	88.4 ± 0.6
POPC:Lyso-16:0-PC 80:20	67 ± 3	18.4 ± 0.3
POPC:Lyso-16:0-PC 85:15	71 ± 3	20.3 ± 0.6
POPC:Lyso-16:0-PC 90:10	75 ± 3	21.7 ± 0.4
POPC:Oleic acid 80:20	95 ± 3	44.8 ± 0.5
POPC:Oleic acid 90:10	90 ± 3	39.9 ± 0.4
POPC:(18:0-24:0)-SM	119 ± 4	43.0 ± 0.7
14:1-14:1-PC (DRPC)	63 ± 5	27.4 ± 0.6
16:1-16:1-PC (DYPC)	82 ± 4	33.6 ± 0.4
18:1-18:1-PC (DOPC)	91 ± 3	41.8 ± 0.6
20:1-20:1-PC (DGPC)	117 ± 4	46.8 ± 0.4
22:1-22:1-PC (DEPC)	136 ± 3	56.4 ± 0.6
12:0-12:0-PC (DLPC)	39 ± 4	15.3 ± 0.6
18:0-18:1-PC (SOPC)	87 ± 3	32.1 ± 0.4
18:2-18:2-PC (DUPC)	88 ± 3	40.5 ± 0.3
18:2-18:3-PC (LLPC)	88 ± 4	37.6 ± 0.3

References

(1) Doktorova, M.; Symons, J. L.; Zhang, X.; Wang, H.-Y.; Schlegel, J.; Lorent, J. H.; Heberle, F. A.; Sezgin, E.; Lyman, E.; Levental, K. R., et al. Cell membranes sustain phospholipid imbalance via cholesterol asymmetry. *bioRxiv* **2023**, 2023–07.

(2) Pogozheva, I. D.; Armstrong, G. A.; Kong, L.; Hartnagel, T. J.; Carpino, C. A.; Gee, S. E.; Picarello, D. M.; Rubin, A. S.; Lee, J.; Park, S.; Lomize, A. L.; Im, W. Comparative molecular dynamics simulation studies of realistic eukaryotic, prokaryotic, and archaeal membranes. *J. Chem. Inf. Comput. Sci.* **2022**, *62*, 1036–1051.

(3) Reinhard, J.; Starke, L.; Klose, C.; Haberkant, P.; Hammarén, H.; Stein, F.; Klein, O.; Berhorst, C.; Stumpf, H.; Sáenz, J. P.; Hub, J.; Schuldiner, M.; Ernst, R. MemPrep, a new technology for isolating organellar membranes provides fingerprints of lipid bilayer stress. *EMBO J.* **2024**, 1–33.

(4) Pluhackova, K.; Horner, A. Native-like membrane models of E. coli polar lipid extract shed light on the importance of lipid composition complexity. *BMC Biol.* **2021**, *19*, 1–22.

(5) Knight, C. J.; Hub, J. S. MemGen: A general web server for the setup of lipid membrane simulation systems. *Bioinformatics* **2015**, *31*, 2897–2899.

(6) Hossein, A.; Deserno, M. Rigidity of Asymmetric and Asymmetrically Stressed Membranes. *Biophys. J.* **2019**, *116*, 89a.

(7) Ting, C. L.; Awasthi, N.; Müller, M.; Hub, J. S. Metastable Prepores in Tension-Free Lipid Bilayers. *Biophys. Rev. Lett.* **2018**, *120*, 128103.

(8) Jo, S.; Kim, T.; Iyer, V. G.; Im, W. CHARMM-GUI: a web-based graphical user interface for CHARMM. *J. Comput. Chem.* **2008**, *29*, 1859–1865.

(9) Jo, S.; Lim, J. B.; Klauda, J. B.; Im, W. CHARMM-GUI Membrane Builder for mixed bilayers and its application to yeast membranes. *Biophys. J.* **2009**, *97*, 50–58.

(10) Abraham, M. J.; Murtola, T.; Schulz, R.; Páll, S.; Smith, J. C.; Hess, B.; Lindahl, E. GROMACS: High performance molecular simulations through multi-level parallelism from laptops to supercomputers. *SoftwareX* **2015**, *1*, 19–25.

(11) Klauda, J. B.; Venable, R. M.; Freites, J. A.; O'Connor, J. W.; Tobias, D. J.; Mondragon-Ramirez, C.; Vorobyov, I.; MacKerell Jr, A. D.; Pastor, R. W. Update of the CHARMM all-atom additive force field for lipids: validation on six lipid types. *J. Phys. Chem. B* **2010**, *114*, 7830–7843.

(12) Lim, J. B.; Rogaski, B.; Klauda, J. B. Update of the cholesterol force field parameters in CHARMM. *J. Phys. Chem. B* **2012**, *116*, 203–210.

(13) Klauda, J. B.; Monje, V.; Kim, T.; Im, W. Improving the CHARMM force field for polyunsaturated fatty acid chains. *J. Phys. Chem. B* **2012**, *116*, 9424–9431.

(14) Venable, R. M.; Sodt, A. J.; Rogaski, B.; Rui, H.; Hatcher, E.; MacKerell, A. D.; Pastor, R. W.; Klauda, J. B. CHARMM all-atom additive force field for sphingomyelin: elucidation of hydrogen bonding and of positive curvature. *Biophys. J* **2014**, *107*, 134–145.

(15) West, A.; Zoni, V.; Teague Jr, W. E.; Leonard, A. N.; Vanni, S.; Gawrisch, K.; Tristram-Nagle, S.; Sachs, J. N.; Klauda, J. B. How do ethanolamine plasmalogens contribute to order and structure of neurological membranes? *J. Phys. Chem. B* **2020**, *124*, 828–839.

(16) Jorgensen, W. L.; Chandrasekhar, J.; Madura, J. D.; Impey, R. W.; Klein, M. L. Comparison of Simple Potential Functions for Simulating Liquid Water. *J. Chem. Phys.* **1983**, *79*, 926–935.

(17) Bjelkmar, P.; Larsson, P.; Cuendet, M. A.; Hess, B.; Lindahl, E. Implementation of the CHARMM force field in GROMACS: analysis of protein stability effects from correction maps, virtual interaction sites, and water models. *J. Chem. Theory Comput.* **2010**, *6*, 459–466.

(18) Bernetti, M.; Bussi, G. Pressure control using stochastic cell rescaling. *J. Chem. Phys.* **2020**, *153*, 114107.

(19) Essmann, U.; Perera, L.; Berkowitz, M. L.; Darden, T.; Lee, H.; Pedersen, L. G. A Smooth Particle Mesh Ewald Method. *J. Chem. Phys.* **1995**, *103*, 8577–8593.

(20) Miyamoto, S.; Kollman, P. A. Settle: An Analytical Version of the SHAKE and RATTLE Algorithm for Rigid Water Models. *J. Comput. Chem.* **1992**, *13*, 952–962.

(21) Hess, B. P-LINCS: A Parallel Linear Constraint Solver for Molecular Simulation. *J. Chem. Theory Comput.* **2007**, *4*, 116–122.

(22) Balusek, C.; Hwang, H.; Lau, C. H.; Lundquist, K.; Hazel, A.; Pavlova, A.; Lynch, D. L.; Reggio, P. H.; Wang, Y.; Gumbart, J. C. Accelerating membrane simulations with hydrogen mass repartitioning. *J. Chem. Theory Comput.* **2019**, *15*, 4673–4686.

(23) Torrie, G. M.; Valleau, J. P. Monte Carlo free energy estimates using non-Boltzmann sampling: Application to the sub-critical Lennard-Jones fluid. *Chem. Phys. Letters* **1974**, *28*, 578–581.

(24) Hub, J. S. Joint Reaction Coordinate for Computing the Free-Energy Landscape of Pore Nucleation and Pore Expansion in Lipid Membranes. *J. Chem. Theory Comput.* **2021**, *17*, 1229–1239.

(25) Hub, J. S.; Awasthi, N. Probing a continuous polar defect: A reaction coordinate for pore formation in lipid membranes. *J. Chem. Theory Comput.* **2017**, *13*, 2352–2366.

(26) Awasthi, N.; Hub, J. S. In *Biomembrane Simulations: Computational Studies of Biological Membranes*; Berkowitz, M. L., Ed.; CRC Press, 2019; pp 109–124.

(27) Kasparyan, G.; Hub, J. S. Molecular Simulations Reveal the Free Energy Landscape and Transition State of Membrane Electroporation. *Phys. Rev. Lett.* **2024**, *132*, 148401.

(28) Kasparyan, G.; Hub, J. S. Equivalence of charge imbalance and external electric fields during free energy calculations of membrane electroporation. *bioRxiv* **2023**, 2023–01.

(29) Kumar, S.; Bouzida, D.; Swendsen, R.; Kollman, P.; Rosenberg, J. THE weighted histogram analysis method for free-energy calculations on biomolecules. I. The method. *J Comput Chem* **1992**, *13*.

(30) Hub, J. S.; De Groot, B. L.; van der Spoel, D. g_wham - A Free Weighted Histogram Analysis Implementation Including Robust Error and Autocorrelation Estimates. *J. Chem. Theory Comput.* **2010**, *6*, 3713–3720.

(31) Nosé, S. A molecular dynamics method for simulations in the canonical ensemble. *Mol. Phys.* **1984**, *52*, 255–268.

(32) Hoover, W. G. Canonical dynamics: Equilibrium phase-space distributions. *Phys. Rev. A* **1985**, *31*, 1695.

(33) Parrinello, M.; Rahman, A. Crystal structure and pair potentials: A molecular-dynamics study. *Phys. Rev. Lett.* **1980**, *45*, 1196.

(34) Allolio, C.; Harries, D. Calcium ions promote membrane fusion by forming negative-curvature inducing clusters on specific anionic lipids. *ACS Nano* **2021**, *15*, 12880–12887.

(35) Goetz, R.; Lipowsky, R. Computer simulations of bilayer membranes: self-assembly and interfacial tension. *J. Chem. Phys.* **1998**, *108*, 7397–7409.

(36) Sega, M.; Fábián, B.; Jedlovszky, P. Pressure profile calculation with mesh Ewald methods. *J. Chem. Theory Comput.* **2016**, *12*, 4509–4515.

(37) Konar, S.; Arif, H.; Allolio, C. Mitochondrial Membrane Model: Lipids, Elastic Properties and the Changing Curvature of Cardiolipin. *Biophys. J.* **2023**, *122*, 4274–4287.

(38) Schofield, P.; Henderson, J. R. Statistical mechanics of inhomogeneous fluids. *Proc. R. Soc. Lond. A* **1982**, *379*, 231–246.

(39) Allolio, C.; Haluts, A.; Harries, D. A local instantaneous surface method for extracting membrane elastic moduli from simulation: Comparison with other strategies. *Chem. Phys.* **2018**, *514*, 31–43.

(40) Johner, N.; Harries, D.; Khelashvili, G. Implementation of a methodology for determining elastic properties of lipid assemblies from molecular dynamics simulations. *BMC Bioinform.* **2016**, *17*, 1–11.

(41) Khelashvili, G.; Kollmitzer, B.; Heftberger, P.; Pabst, G.; Harries, D. Calculating the bending modulus for multicomponent lipid membranes in different thermodynamic phases. *Journal of chemical theory and computation* **2013**, *9*, 3866–3871.

(42) Szleifer, I.; Kramer, D.; Ben-Shaul, A.; Gelbart, W. M.; Safran, S. A. Molecular theory of curvature elasticity in surfactant films. *J. Chem. Phys.* **1990**, *92*, 6800–6817.

(43) Hamm, M.; Kozlov, M. Elastic energy of tilt and bending of fluid membranes. *Eur. Phys. J. E* **2000**, *3*, 323–335.

(44) Venable, R. M.; Brown, F. L.; Pastor, R. W. Mechanical properties of lipid bilayers from molecular dynamics simulation. *Chem. Phys. Lipids* **2015**, *192*, 60–74.

(45) Pöhnl, M.; Trollmann, M. F.; Böckmann, R. A. Nonuniversal impact of cholesterol on membranes mobility, curvature sensing and elasticity. *Nat Commun* **2023**, *14*, 8038.

(46) Portet, T.; Dimova, R. A new method for measuring edge tensions and stability of lipid bilayers: effect of membrane composition. *Biophys. J.* **2010**, *99*, 3264–3273.

(47) Chernomordik, L.; Kozlov, M.; Melikyan, G.; Abidor, I.; Markin, V.; Chizmadzhev, Y. A. The shape of lipid molecules and monolayer membrane fusion. *Biochim. Biophys. Acta, Biomembr.* **1985**, *812*, 643–655.

(48) Karatekin, E.; Sandre, O.; Guitouni, H.; Borghi, N.; Puech, P.-H.; Brochard-Wyart, F. Cascades of transient pores in giant vesicles: line tension and transport. *Biophys. J.* **2003**, *84*, 1734–1749.

(49) Zhelev, D. V.; Needham, D. Tension-stabilized pores in giant vesicles: determination of pore size and pore line tension. *Biochim. Biophys. Acta, Biomembr.* **1993**, *1147*, 89–104.

(50) Tazawa, K.; Yamazaki, M. Effect of monolayer spontaneous curvature on constant tension-induced pore formation in lipid bilayers. *J. Chem. Phys.* **2023**, *158*, 081101.

(51) Lira, R. B.; Leomil, F. S.; Melo, R. J.; Riske, K. A.; Dimova, R. To close or to collapse: the role of charges on membrane stability upon pore formation. *Adv. Sci.* **2021**, *8*, 2004068.
